# Lockjaw

**Published:** 1893-05

**Authors:** 


LOCKJAW.—A long series of experiments, illustrating one of the favorite methods of modern science, has proved the terrible disease of lockjaw, or tetanus, to be curable. Until last year it was fatal in nine cases out of ten.

Seven years ago Nicolaier, a young German doctor, found all the symptoms of tetanus in mice under whose skin he had placed minute portions of dirt from dirt of open fields. He found that pus from the wounds gave tetanus to mice or rabbits inoculated with it, and that this pus contained a peculiar bacillus shaped like a drumstick.

Kitasato, a young Japanese student with Doctor Koch, of Berlin, succeeded in 1889 in separating the bacillus from the pus. So did two Italian students, one of them a woman. They found that the minutest drop of the "culture," when placed under the skin of animals, gave lockjaw, and they showed that this was because the drumstick-shaped bacilli contained a virulent poison, separable from the bacilli.

It was also shown that tetanus is always due to the same bacillus, and that this is found everywhere in the surface soil of streets or fields.

In 1890 Kitasato and another student discovered that rabbits treated with tri chloride of iodine were almost perfectly "immune," or safe from lockjaw, and that their blood injected into a normal rabbit protected it from the malady. Moreover, the "immune" animal blood was found to cure far advanced lockjaw in white mice.

The two Italian doctors, who had shared Kitasato's first discovery, announced further that they had made dogs proof against lockjaw by inoculating them gradually with violent tetanus "cultures," and that from the blood of such "immune" dogs they had extracted an albuminous substance which would destroy the drumstick-shaped bacillus wherever found. From this albumen they finally made a fine powder which retains its curative properties for months.

The next step was to try the cure on human sufferers from lockjaw. In 1891 came twelve opportunities to do so, and in every case, some of them severe in the extreme, the patient was wholly cured.

Thus the result of sacrificing a limited number of the lower creatures is the saving of countless human lives through all future time.—*The Youth's Companion.*

